# Patient therapy outcome modeling in cancer organoids is improved by cancer‐associated fibroblasts and organoid assembly convolution

**DOI:** 10.1002/1878-0261.70282

**Published:** 2026-06-05

**Authors:** Marcin Grochowski, Liudmyla Dolinchuk, Michał Jerzak, Albert Gandurski, Tomasz Grochowski, Weronika Wojtyś, Maciej Zadrożny, Wojciech Kaźmierczak, Małgorzata Lenarcik, Marta Matejak‐Górska, Radosław Samsel, Tomasz Olesiński, Dawid Walerych

**Affiliations:** ^1^ Mossakowski Medical Research Institute PAS Warsaw Poland; ^2^ Doctoral School of Translational Medicine Center for Postgraduate Medical Education Warsaw Poland; ^3^ Medical University of Warsaw Poland; ^4^ Maria Skłodowska‐Curie National Research Institute of Oncology Warsaw Poland; ^5^ Clinical Department of Gastroenterological Surgery and Transplantology National Medical Institute of the Ministry of the Interior and Administration Warsaw Poland

**Keywords:** organoids, microenvironment, cancer‐associated fibroblasts, pancreatic cancer, colon cancer, gastric cancer

## Abstract

Patient‐derived organoids (PDOs) are becoming established as preclinical models for predicting therapeutic responses in cancer, yet their clinical accuracy remains limited by the insufficient representation of the tumor microenvironment and a reliance on static viability readouts. Here, we utilized a living biobank of 30 histopathologically and genetically characterized PDOs, alongside a microenvironment‐derived from pancreatic, colon, and gastric cancers, to systematically evaluate their ability to respond to standard‐of‐care or experimental therapies and model patient outcomes. We assessed the impact of incorporating tissue‐matched cancer‐associated fibroblasts (CAFs) on treatment responses, finding that their presence not only increased chemoresistance in viability assays but significantly improved patient outcome prediction. To further enhance this predictive accuracy, we developed the Organoid Convolution Assay (OCA), a live‐cell imaging‐based approach that quantitatively captures dynamics of cell migration, clustering, and assembly during organoid formation. Mathematical modeling of these parameters enabled the significant stratification of donor tumors by stage (T0–T2 vs. T3–T4) and the prediction of patient clinical outcomes. Together, our findings demonstrate that incorporating either tumor microenvironment components or dynamic organoid assembly metrics improves the clinical relevance of PDO‐based models.

## Graphical abstract

Patient‐derived organoids (PDOs) from pancreatic, colorectal, and gastric cancers were used to evaluate standard and experimental therapies. Incorporating cancer‐associated fibroblasts (CAFs) into organoid cultures improved patient therapy outcome prediction. Novel Organoid Convolution Assay (OCA) captured dynamic organoid assembly that allowed sample stratification by tumor stage and clinical outcome. In conclusion, microenvironment integration or organoid dynamics readouts increased PDOs' clinical relevance.

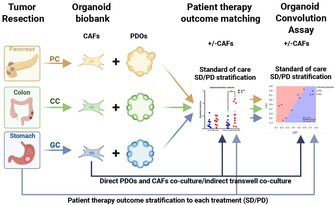

**Abbreviations**
5‐FU: 5‐fluorouracil
ASMA: Alpha‐Smooth Muscle Actin
AUC: Area under the curve
BME: Basement Membrane Extract
CAF(s): Cancer‐associated fibroblast(s)
CC: Colon cancer
CNV(s): Copy number variation(s)
FAP: Fibroblast activation protein
GC: Gastric cancer
H&E: Hematoxylin and eosin
IL‐6: Interleukin‐6
MAF: Mutation Annotation Format
OCA: Organoid Convolution Assay
PC: Pancreatic cancer
PD: Progressive disease
PDA: Pancreatic ductal adenocarcinoma
PDO(s): Patient‐derived organoid(s)
RECIST: Response evaluation criteria in solid tumors
SD: Stable disease
WES: Whole‐exome sequencing
WHO: World Health Organization

## 1 Introduction

Cancer remains a widespread disease, with a constant need for new preclinical models and personalized therapies. Pancreatic ductal adenocarcinoma (PDA), colorectal cancer (CC), and gastric cancer (GC) are either among the most lethal or most frequent cancer types [1, 2, 3]. Given their high prevalence, poor prognosis, and limited treatment options for these cancers, there is a pressing need for models that accurately recapitulate tumor biology and predict patient‐specific responses. In this context, patient‐derived organoids (PDOs) are increasingly recognized as important research models, representing tumor heterogeneity [4, 5, 6]. Compared with traditional cell lines, PDOs allow for a clinically relevant testing of therapeutic responses and hold promise for advancing personalized medicine approaches [7, 8]. Recent studies have shown that patient‐derived cancer samples can be grown as organoids efficiently and accurately enough to be potentially useful for clinical decision‐making. For instance, the drug sensitivity of 12 patient‐derived PDA organoids tested against a series of standard‐of‐care therapeutic protocols reliably predicted clinical responses to treatment in individual patients, with a clinical correlation observed based on an AUC‐based threshold [9]. In the TUMOROID study, PDOs from 29 metastatic CC patients were tested *in vitro* and compared with clinical response data for irinotecan‐based therapies; the PDOs correctly predicted patient responses in more than 80% of cases [10]. Similarly, the drug responses of organoids derived from 12 GC patients to oxaliplatin and 5‐FU were validated against corresponding clinical outcomes, with 11 of 12 PDOs showing concordant responses [11].

As the representative examples listed above demonstrate, studies comparing organoids and patients are still limited in the number of patients included. This is one of the main reasons why organoid‐based companion diagnostic protocols have not yet proceeded beyond clinical trials, as there is a need for more cumulative evidence supporting this approach for each disease and therapy protocol. Additionally, monoculture PDO models do not fully capture the complexity of the tumor microenvironment, which may affect the accuracy of companion diagnostics. Stromal and immune cells strongly affect tumor progression and treatment resistance [12]. Among them, cancer‐associated fibroblasts (CAFs) play a particularly important role [13, 14]. Recent studies show that CAFs are a key factor in the tumor microenvironment and can trigger acinar‐to‐ductal metaplasia through their secreted factors. This effect is driven by the LAMA5/ITGA4/STAT3 signaling axis, and can be reproduced with CAF‐conditioned media alone [15], while CAF‐secreted factors have also been shown to paracrinally activate STAT3 in carcinoma cells [16]. Several studies have shown that PDOs incorporating patient‐derived CAFs impact the efficiency of tested therapeutic protocols without correlating this result with the patients' clinical response. Schuth et al. established direct 3D co‐cultures of organoids and CAF lines derived from 5 PDA patients and observed significantly reduced chemosensitivity to gemcitabine, 5‐FU and paclitaxel in co‐cultures compared with monocultures [14]. Farin et al. generated a CC organoid–stroma biobank from 30 patients and demonstrated that the incorporation of CAFs markedly altered therapeutic responses, with CMS4 subtype‐derived models (12 patients) showing significantly reduced sensitivity to gefitinib and SN‐38 compared with monocultures, whereas CAF‐dependent effects on 5‐fluorouracil and oxaliplatin were heterogeneous across the cohort (29 patients) [13]. This demonstrates that the introduction of CAFs alters the PDO sensitivity to tested therapies; however, cancer‐ or protocol specificity and the correlation of this effect with patient therapy outcomes, requires further investigation.

Previously, we introduced novel experimental drug combinations and validated them using colon‐ and pancreatic cancer‐derived organoids [17, 18]. In this study, we addressed three further goals using cancer organoid models: first, we compared the PDO sensitivity to our experimental anticancer drug combinations with standard‐of‐care chemotherapies using a characterized, patient‐derived biobank of colon, pancreatic and GC organoid models. Second, we determined whether the introduction of CAFs to organoid cultures affects their response to both standard and experimental anticancer drug combinations, as well as the prediction of donor patient clinical outcomes for matching therapy regimens. Finally, we sought to improve the accuracy of these predictions by introducing the Organoid Convolution Assay (OCA), which allows for the determination of tumor staging and therapy response in patients more accurately than organoid viability measurements, with or without the presence of CAFs. These results contribute to the ongoing worldwide effort to improve the accuracy of the cancer organoid models to provide clinically significant information for personalized medicine.

## 2 Materials and methods

### 2.1 Antibodies

The following antibodies were used in this study: FAP (clone F1A4G); Cell Signaling Technology (Danvers, MA, USA), alpha‐Smooth Muscle Actin (clone D4K9N); Cell Signaling Technology, IL‐6 (clone D5W4V); Cell Signaling Technology, beta‐Actin (clone 8H10D10); Cell Signaling Technology, E‐Cadherin (clone 24E10); Cell Signaling Technology, GATA6 (clone D61E4); Cell Signaling Technology, Keratin 7 (clone RN7); Cell Signaling Technology, Keratin 20 (clone D9Z1Z); Cell Signaling Technology, Alexa Fluor™ 48 secondary antibody; Thermo Fisher Scientific (Waltham, MA, USA), Alexa Fluor™ 546 secondary antibody; Thermo Fisher Scientific, Goat Anti‐Mouse IgG (H + L)‐HRP Conjugate; Bio‐Rad, and Goat Anti‐Rabbit IgG (H + L)‐HRP Conjugate; Bio‐Rad.

### 2.2 Drugs

Thw following drugs were used in this study: Selinexor (HY‐17536); MedChemExpress (Monmouth Junction, NJ, USA), MKT‐077 (HY‐15096); MedChemExpress, CB‐6644 (HY‐114429); MedChemExpress, Carfilzomib (HY‐10455); MedChemExpress, VER‐155008 (HY‐10941); MedChemExpress, Nelfinavir (HY‐15287); MedChemExpress, Nab‐paclitaxel (HY‐P99974); MedChemExpress, Gemcitabine (HY‐17026); MedChemExpress, 5‐Fluorouracil (HY‐90006); MedChemExpress, Oxaliplatin (HY‐17371); MedChemExpress, Irinotecan (HY‐16562); MedChemExpress, Leucovorin (HY‐17556); MedChemExpress, and Docetaxel (HY‐B0011); MedChemExpress.

### 2.3 Collection and preliminary characterization of clinical samples

All patient‐derived samples were collected based on ethical committee approvals (no.: 109/2016) from the National Medical Institute of the Ministry of the Interior and Administration in Warsaw, Poland (with 2020 updates including NIO Warsaw in the study; samples collected January 2019 to December 2022) and (no.: 55/2023) from the Maria Skłodowska‐Curie National Research Institute of Oncology in Warsaw, Poland (NIO Warsaw) (samples collected from August 2023 to October 2025). Written informed consent for the research use of collected tissues was obtained from all patients, and samples were anonymized. The study methodologies conformed to the standards set by the Declaration of Helsinki. After surgical resection, samples were subjected to preliminary histopathological assessment and subsequently stored in culture medium at 4 °C for organoid culture establishment. Due to clinical material amount availability in most cases the whole ressected tumor tissue was used to establish organoid cultures and perform parallel histopathologic examination of fixed fragments, with no material remaining for additional frozen tissue storage and analysis. No additional omics analysis was performed in the hospital units and was otherwise limited due to funding constraints.

### 2.4 Establishment and culture of colon tumor organoids

Primary tumor organoid cultures were established using a modified version of the protocol previously described in detail in Boj et al. [19], these modifications were described in detail in Oroń et al. [18]. All experiments were conducted exclusively with mycoplasma‐free cells, verified through routine testing every 6 months.

### 2.5 Establishment and culture of pancreatic tumor organoids

Pancreatic tumor organoids were established in a manner similar to colon organoids based on (2016) [19], with the addition of 10 nm human Gastrin I (Tocris, Bristol, UK) and the exclusion of SB202190 from the culture medium.

### 2.6 Establishment and culture of gastric tumor organoids

Gastric tumor organoids were generated in a manner similar to colon organoids based on Boj et al. (2016) [19], with addition of 10 nm human Gastrin I (Tocris, UK) and the exclusion of SB202190 from the culture medium.

### 2.7 Isolation and culture of colon, pancreatic and gastric cancer‐associated fibroblasts (CAFs)

Primary CAFs were isolated from the previously established colon, pancreatic and gastric tumor organoid cultures. Immediately after the first passage of the organoid cultures, DMEM medium (Gibco, Brooklyn, NY, USA) supplemented with 10% fetal bovine serum and penicillin–streptomycin antibiotics was added to the remaining wells still containing cells exhibiting strong adhesion to the well coating. Cells were cultured in the aftermentioned medium until reaching 100% confluency and subsequently passaged into cell culture dishes for expansion. The resulting CAF lines were expanded in DMEM medium, which was changed every 3–4 days. CAF lines were frozen and stored in liquid nitrogen for future use.

### 2.8 Hematoxylin and eosin staining of human colon, pancreatic and gastric organoids and patient‐derived cancer tissues

PDOs were fixed in 4% paraformaldehyde (PFA) for 2 h at 4 °C. After fixation, samples were rinsed in phosphate‐buffered saline (0.1 m PBS, pH 7.4) and incubated in a 30% sucrose solution at 4 °C until infiltration was complete (~ 12–24 h). The organoids were then transferred into Tissue Freezing Medium (Leica Biosystems, Nussloch, Germany). To ensure positioning at the bottom of the embedding mold, samples were centrifuged at 100 × ***g*** for 3 min using a swinging‐bucket centrifuge (Thermo Scientific, Waltham, MA, USA). The embedded samples were snap‐frozen on dry ice and stored at −80 °C until further processing. Cryosections (10 μm) were prepared using a cryostat (Leica CM1850, Leica Biosystems, Germany) and stained with hematoxylin and eosin (H&E) solution. Stained organoids were imaged using a Zeiss Axiovert 135 microscope (Carl Zeiss, Germany) with a Nikon camera (DS‐Ri2); NIS software (Nikon, Japan) was used for capturing the images. PDOs were fixed and processed according to standard formalin‐fixed paraffin‐embedded protocols and observed using light microscopy. The histological type and the tumor grade were classified based on the WHO classification, while the tumor stage was determined according to the 8th edition of AJCC/UICC pTNM [20].

### 2.9 Western blot

Western blot was performed as described previously [18]. The list of used antibodies is presented in Section 2.1.

### 2.10 DNA isolation

DNA was extracted from cell lines using the Tissue DNA Purification Kit (EURx, Poland), following the manufacturer's instructions. DNA quantity and quality were assessed utilizing a NanoDrop spectrophotometer (Thermo Fisher Scientific, USA).

### 2.11 Whole‐exome sequencing and analysis

Library preparation was performed using the Twist Bioscience Exome Kit in accordance with the manufacturer's instructions. Sequencing was performed using an Illumina NovaSeq6000 in a paired‐end 2 × 100‐bp mode. Demultiplexing of the raw sequencing reads was performed with Illumina bcl2fastq (version 2.20). Adapters were trimmed with Skewer (version 0.2.2). Sequencing data analysis was performed using the Illumina DRAGEN platform (version 4.2.4). Reads were mapped to the reference genome GRCh38 and duplicates were marked. Calling of small variants was performed using the DRAGEN somatic tumor‐only pipeline with default parameters. Copy number variations (CNVs) were called using a Panel of Normals (PoN). Only high‐quality reads were included in the further analysis. The resulting VCF files were converted to the Mutation Annotation Format (MAF) using vcf2maf (version 1.6.22), which utilized the Ensembl Variant Effect Predictor 115 release to generate functional annotations. Common variants were excluded if they exceeded an allele frequency threshold of 0.04% in any gnomAD sub‐population. Oncoplots and multi‐panel summary plots were generated using the maftools and ComplexHeatmap packages, R version 4.5.2 and RStudio [21, 22, 23].

### 2.12 Drug sensitivity assays in organoids

Forty microliters of ice‐cold Basement Membrane Extract (BME) type 2 (R&D Systems, Bio‐Techne, USA) were added to 30 wells of a 96‐well plate. The plate was then placed in a 37 °C, 5% CO_2_ incubator for 30 min. Next, organoids were dissociated with TrypLE (Gibco) for 15 min at 37 °C and cells were counted using a Countess 2 Automated Cell Counter (Thermo Fisher Scientific, USA). A total of 100 000 cells suspended in the 100 μL of culture medium were added to each well. After 48 h, the drugs were added to the culture medium at the concentrations indicated in the figures. For each drug sensitivity assay, three wells were used as technical replicates. The organoids were incubated with the drugs for 72 h; then, cell viability was measured using the ATPlite One Step Reagent (PerkinElmer Inc., Shelton, CT, USA).

### 2.13 Drug sensitivity assays in organoids‐CAFs direct co‐cultures

Drug sensitivity assays in direct co‐cultures were performed in a manner similar to the previously described protocol for drug testing in organoid monocultures (Section 2.12), but with the addition of CAFs. Briefly, 40 μL of ice‐cold BME type 2 (R&D Systems, Bio‐Techne, USA) were added to 30 wells of a 96‐well plate. Plates were then placed in a 37 °C, 5% CO_2_ incubator for 30 min. Then, organoids and CAFs were dissociated with TrypLE (Gibco) and counted. To each well, 100 000 organoid cells and 100 000 CAFs suspended in 100 μL of organoid culture medium (OCM) were added. After 48 h, the drugs were added to the OCM at the concentrations indicated in the figures. For each drug sensitivity assay, three wells were used as technical replicates. The co‐cultures were incubated with the drugs for 72 h; then, cell viability was measured using the ATPlite One Step Reagent (PerkinElmer Inc., USA).

### 2.14 Drug sensitivity assays in organoids‐CAFs indirect co‐cultures

Drug sensitivity assays in indirect co‐cultures were conducted using cell culture inserts. PDOs and CAFs were dissociated and counted as previously described. For each drug assay, 300 000 organoid cells were embedded in 90 μL of BME type 2 (R&D Systems, Bio‐Techne, USA). Three 30 μL BME domes were placed in a single well of a 24‐well plate and placed in a 37 °C, 5% CO_2_ incubator for 30 min. Organoid culture medium was added and a culture insert was placed into the well. Then, 300 000 CAFs suspended in 200 μL OCM were added to the insert. After 48 h, the drugs were added to the OCM at the concentrations indicated in the figures. The co‐culture was incubated with the drugs for 72 h, then cell viability was measured using the ATPlite One Step Reagent (PerkinElmer Inc., USA).

### 2.15 Light microscopy

The morphology of cancer PDOs cultured for 8–14 days was observed using a bright‐field light microscope (Leica, Germany) at 10× and 20× magnification.

### 2.16 Immunofluorescence staining of human colon, pancreatic, and gastric organoids

PDOs were grown in chamber slides (Ibidi, Germany). After 7 days of culture, the organoids were fixed in 4% paraformaldehyde for 20 min, followed by three washes in PBS with glycine. Cells were permeabilized using 0.5% Triton X‐100 in PBS for 10 min and nonspecific binding sites were blocked with 3% BSA in PBS. The following day, samples were incubated at 4 °C overnight with a primary antibody against E‐Cadherin (Cell Signaling Technology, USA, clone 24E10), then washed, and probed with an Alexa Fluor 555 secondary antibody (Life Technologies, Waltham, MA, USA, A21428), also overnight at 4 °C. Nuclear staining was performed with 0.5 ng/mL DAPI. Imaging was performed using a Carl Zeiss LSM 780 confocal microscope (Carl Zeiss, Oberkochen, Germany).

### 2.17 Organoid Convolution Assay

Human cancer organoids were enzymatically dissociated (15 min using TrypLE (Gibco) with 250 RCF shaking) into a single‐cell suspension. Subsequently, dissociated cells were plated on top of BME type 2 (R&D Systems, Bio‐Techne, Minneapolis, MN, USA), with which the wells of imaging plates (μ‐Slide 15 Well 3D Glass Bottom (Ibidi, Gräfelfing, Germany)) were covered. After 30 min at 37 °C in a cell culture incubator, the cells were covered with OCM supplemented with 10% BME. Imaging and recording of cell migration and organoid reassembly were conducted using a Zeiss Cell Observer SD light microscopy system (Carl Zeiss, Germany). Raw data extraction was performed using the ImageJ software and the TrackMate plugin. A set of measurement parameters describing the process of organoid reassembly from dissociated cells was established. These parameters are:

DAA—The relative change in the average surface area of the object [μm^2^] during the experiment, calculated as the ratio of the difference between the ‘Average surface area of the object in the first frame’ and the ‘Average surface area of the object in the last frame’ to the ‘Average surface area of the object in the first frame’.

DV—A measure of increased object motility [μm/s], calculated as the ratio of the ‘Average speed of objects whose speed exceeds the median’ to the ‘Average distance to the nearest object in the first frame’.

DC—The relative change in the number of cells (with a diameter smaller than 9.5 μm—an experimentally determined threshold for pancreatic, gastric, and CCs, above which the objects are considered small organoids rather than single cells) during the experiment. This is calculated per organoid as the ratio of the difference between the ‘Number of cells in the first frame’ and the ‘Number of cells in the last frame’ to the product of the ‘Number of cells in the first frame’ and the ‘Number of organoids in the last frame’. If the number of organoids in the last frame is 0, a value of 1 is used instead.

ANND‐S—Describes the process of clustering or dispersal of cells and organoids within the observation space, based on the slope of the regression line of changes in the average distance to the nearest neighboring object over the course of the experiment.

Next, the described parameters are used to calculate the LS1 and LS2 estimators. LS1 represents the estimator of ‘grade’ and LS2 is the estimator of ‘metastatic potential’, both are defined as discrete and binary, that is, having values of +1 (presence of the feature) or −1 (absence of the feature). These are calculated using the linearized maximum likelihood method—a multidimensional least squares approach. The measurement parameters obtained from reference (calibration) experiments (DAA, DC, ANND‐S, DV) and the expected values of the LS1 and LS2 estimators (+1, −1 values) are processed using the LSQ_LINEAR procedure from the OPTIMIZE module of the SCIPY library (https://scipy.org) in the Python programming language (https://python.org). The resulting coefficients for the LS1 and LS2 estimation expressions provide the best fit to the characteristics (‘grade’ and ‘metastatic potential’) of the reference experiments, yielding the following Eqs. LS1 = 1.440 * [DAA]−7065.0 * [DV]−0.584 * [DC]−10.7525 * [ANND‐S] + 1.218 and LS2 = −0.4951 * [DAA] + 1080.0 * [DV] + 0.6254 * [DC]−5.6675 * [ANND‐S] + 0.4029. The LS1 and LS2 estimators allowed us to cluster our experimental data for each PDO population into separate groups describing the clinical outcomes of oncological patients (during first 9 months of their adjuvant chemotherapy): progressive disease (PD) and stable disease (SD). The computational workflow is also described in the Supporting information. Patent applications for OCA are submitted.

### 2.18 Quantification and statistical analysis

All data are presented as the mean ± standard deviation (SD) or the standard error of the mean (SEM), as indicated in the figure legends. Details regarding the statistical tests applied are provided in the figure legends. Statistical analyses were conducted using GraphPad Prism version 8.0.2, JASP 0.95.4 (for logistic regression classification and MANOVA) and R version 4.5.2 utilizing stats and tidyverse packages (for Mann–Whitney U‐tests and hierarchical clustering).

## 3 Results

### 3.1 Generation of a living, patient‐derived organoid biobank for pancreatic, colon, and gastric cancers

**Fig. 1:**
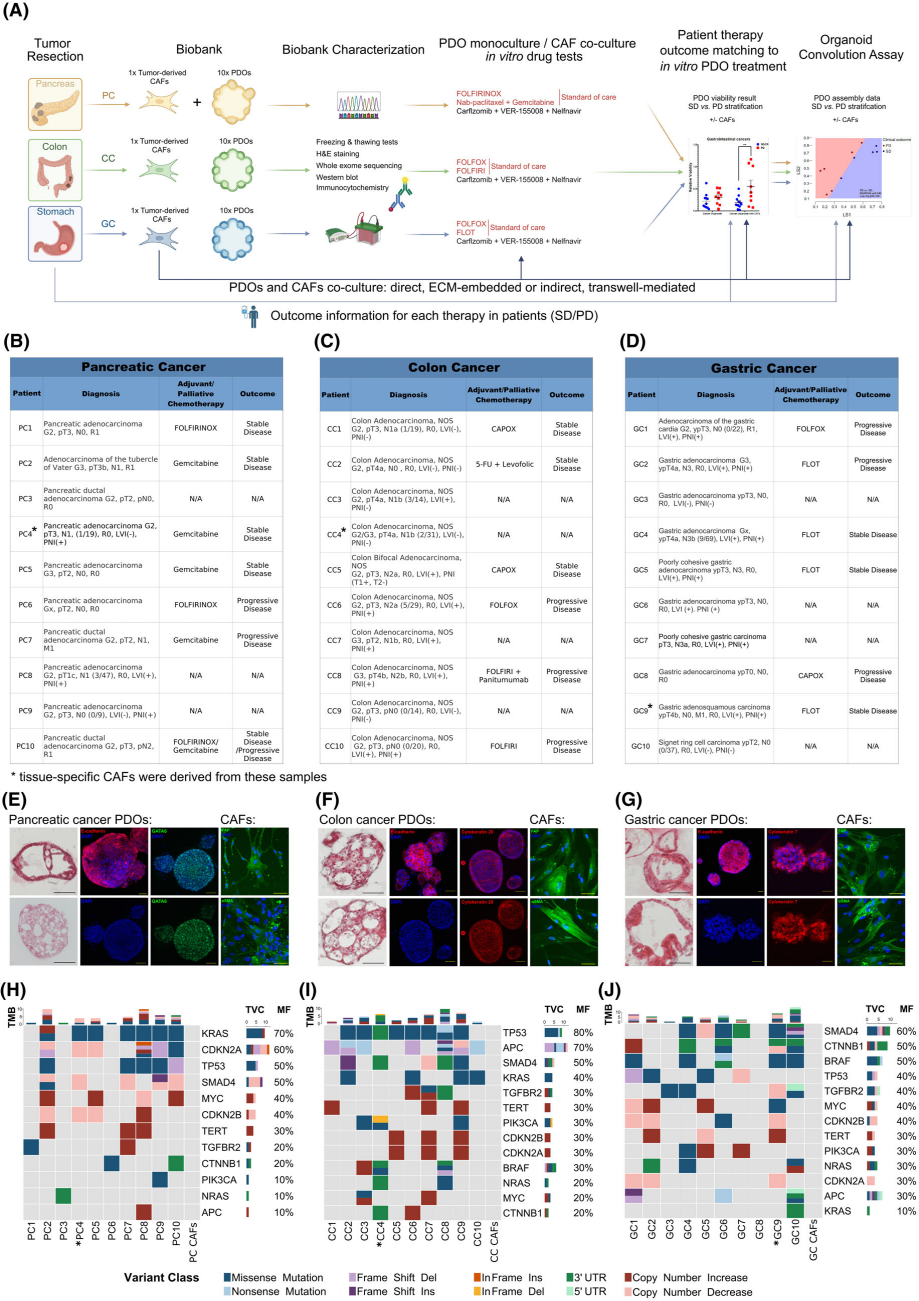
Characterization of a patient‐derived living pancreatic, colon, and gastric cancer organoid biobank. (A) Experimental flowchart of biobank establishment and characterization, followed by experimental procedures for drug and microenvironment testing. CAFs, cancer‐associated fibroblasts; CC, colon cancer; ECM, extracellular matrix; GC, gastric cancer; H&E, hematoxylin and eosin; PC, pancreatic cancer; PD, progressive disease; PDOs, patient‐derived organoids; SD, stable disease. (B–D) Tables summarizing patient diagnosis, treatment and clinical outcome for patient‐derived organoids (PDOs) of each indicated cancer type. CAFs—cancer‐associated fibroblasts. (E–G) Organoid morphology and markers: left panels—bright‐field microscopy images of two representative H&E‐stained pancreatic, colon, and gastric cancer patient‐derived organoids (PDOs), respectively. Scale bar, 60 μm; middle panels—confocal microscopy images of pancreati,c, colon and gastric cancer PDOs organoids, respectively, stained for nuclei (DAPI‐blue) and E‐cadherin (red) or for nuclei (DAPI‐blue) and one of the following: GATA6 (green), Keratin 20 (red) or Keratin 7 (red). Scale bar, 100 μm. Right panels—confocal microscopy images of cancer‐associated fibroblast (CAF) cultures stained for nuclei (DAPI‐blue) and FAP or alpha‐SMA (green). Scale bar, 100 μm. All photographs are representative to *n* = 3 experiment replicates. (H–J) Oncoplots depicting genetic alterations detected by whole‐exome sequencing in pancreatic, colon, and gastric cancer organoids, respectively, and CAFs, for 12 genes chosen based on the cancer driver census in the TCGA and COSMIC databases. Variant types are color‐coded according to the legend at the bottom. Each column represents a single organoid or CAF line, while the top row graph displays its total mutational burden (TMB). Each row corresponds to a selected gene, while the side bar graph displays the number and distribution of its genetic alterations, excluding CAFs (TVC—Total Variant Count, MF—mutation frequency).

To develop a patient‐derived, resection‐based PDO biobank, we collected, processed, established, and selected as stably biobanked 10 assay‐ready organoid cultures of each: pancreatic, colon, and gastric cancers (Fig. 1A). The selection of assay‐ready cultures was primarily based on their ability to successfully undergo a cycle of freezing and thawing. Further, we histopathologically characterized the donor tissues and biobanked PDOs, identifying the histological type and the tumor grade in concordance with WHO classification, as well as tumor stage based on the 8th edition of AJCC/UICC pTNM and the clinical outcome based on Response Evaluation Criteria in Solid Tumors (Fig. 1A–D) [20, 24].

All the pancreatic cancer donor samples were adenocarcinomas, the predominant tumor grade was G2, the predominant T parameter was T3, and in 50% of cases, lymph node infiltration by cancer cells was present. For colon cancer, the samples were adenocarcinomas, the predominant tumor grade was again G2, and predominant T parameter was T3, and in 70% of cases, there was observable cancer cell infiltration to the lymph nodes. In GC, seven samples were adenocarcinomas; for most of the samples, tumor grade was not characterized due to preoperative treatment; the predominant T parameter was T3, and in 40% of cases, there was detectable lymph node infiltration by cancer cells (Fig. 1A–C and Fig. S1A–C). We performed organoid culture assessment using H&E staining to compare them with the original tissue specimens, which revealed cancer‐like cellular features and structures within the organoids, comparable to the donor tissues (Fig. 1E–G, Fig. S1A–G). We also conducted immunofluorescence staining of representative PDOs for markers detectable in each cancer type GATA6 (pancreatic cancer), Keratin 7 (gastric cancer), and Keratin 20 (colon cancer) (Fig. 1E–G).

For each cancer type, we derived CAF cell lines with lower efficiency than PDOs (30–60%) and thus selected a single, stably biobanked, tissue‐specific CAF cell line for further studies. These selected cell lines were validated for CAF markers: presence of α‐Smooth Muscle Actin and FAP via immunofluorescence (Fig. 1E–G). By western blot analysis, FAP was detected in all CAFs as well as in a control normal skin fibroblast cell line, while the level of α‐Smooth Muscle Actin was elevated in CAF lines when compared with normal skin fibroblasts. IL‐6 was absent in all fibroblast cell lines tested, suggesting our CAF lines to be of the myofibroblastic subtype (Fig. S1D) [25]. As a negative control, we tested aforementioned CAF markers in our organoid lines, where they were absent or present at very low levels (Fig. S1D).

To assess the genetic background of the biobank, we performed whole‐exome sequencing (WES) on the organoid samples and CAFs (no tissue was left available for comparative analysis of frozen samples). Data were analyzed according to the GDC DNA‐Seq analysis guidelines [22], revealing mutational and driver oncogene activation profiles, as well as CVNs across the organoid lines (Fig. 1H–J, Fig. S2A–F and Tables S1– S4). These profiles generally matched the earlier reported cancer‐specific genetic profiles [13, 26, 27]. In this way, we built an organoid biobank comprising three digestive system cancer types with 30 representative, assay‐ready cultures characterized histopathologically and genetically, as well as three tissue‐specific CAF cell lines, making the biobank suitable for further analysis.

### 3.2 Comparison of standard and experimental drug combinations in the organoid biobank and the impact of CAFs on the PDO sensitivity to anticancer therapies *in vitro*

We used the organoid biobank to test previously developed experimental therapeutic protocols based on the presence of an activated proteasome in cancer cells or the activation of major driver oncogenes: carfilzomib + VER‐155008 + nelfinavir, and selinexor + MKT‐077 or CB‐6644 [17, 18]. We compared the PDO sensitivity to these protocols with standard‐of‐care chemotherapies (FOLFIRINOX and nab‐paclitaxel + gemcitabine for pancreatic cancer, FOLFOX and FOLFIRI for colon cancer, FOLFOX and FLOT for gastric cancer) [28, 29, 30]. For standard‐of‐care treatments, we kept the molar ratio of components similar to the mass ratio of components used clinically. The drugs were added 48 h after seeding the organoid cells, and the relative viability (measured by the ATP level‐based assay) was measured 72 h after drug addition. We also performed sensitivity/resistance significance analysis for PDO monocultures and PDO‐CAF co‐cultures in relation to the driver mutations found in the WES analysis of the PDOs, revealing possible links of selected sensitivities to *SMAD4* mutations and resistances to *CDKN2A/B* and *TP53* mutations (Fig. S2G–I).

**Fig. 2:**
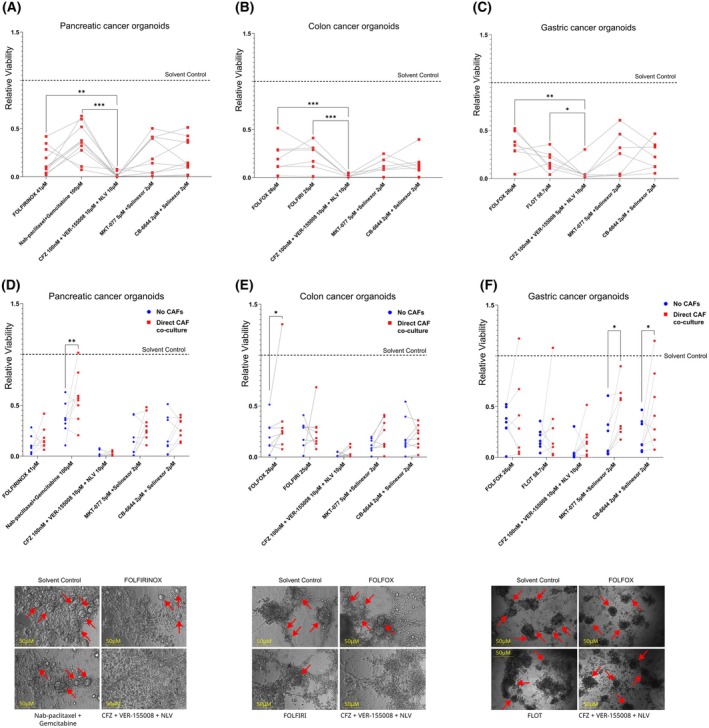
Comparison between standard and experimental drug combinations in the organoid biobank and the impact of cancer‐associated fibroblasts (CAFs) on the patient‐derived organoid (PDO) sensitivity to anticancer therapies *in vitro* (A–C) Relative viability of patient‐derived pancreatic, colon, and gastric cancer organoids, respectively, treated with FOLFIRINOX; nab‐paclitaxel and gemcitabine; FOLFOX; FOLFIRI; FLOT; carfilzomib (CFZ) with VER‐155008 and nelfinavir (NLV); MKT‐077 with selinexor; and CB‐6644 with selinexor using indicated concentrations. Viability was measured using ATPlite 72 h after drug addition to the culture. Each symbol indicates viability of a single patient‐derived organoid culture (for pancreatic cancer *n* = 9 individual patients, for colon cancer *n* = 7 individual patients, for gastric cancer *n* = 7 individual patients; each is an average of three technical replicates) relative to the control, with horizontal lines linking the same sample under different treatments. Dashed lines indicate average, normalized solvent control levels. Statistical analysis of difference between sample‐matched PDO populations was performed using Friedman test, *P < 0.05, **P < 0.01, ***P < 0.001. (D–F) Comparison of the relative viability results for pancreatic, colon, and gastric cancer organoids, respectively, co‐cultured directly with CAFs or as a monoculture, treated with indicated drug compositions at indicated concentrations. Viability was measured using ATPlite 72 h after drug addition to the culture. Dashed lines indicate average, normalized solvent control levels. Each symbol indicates viability of a single patient‐derived PDO (for pancreatic cancer *n* = 8 individual patients, for colon cancer *n* = 8 individual patients, for gastric cancer *n* = 8 individual patients; each is an average of three technical replicates) relative to the control, with horizontal lines linking the same sample in a monoculture and a direct CAF co‐culture. Dashed lines indicate average, normalized solvent control levels. Statistical analysis of difference between sample‐matched PDO populations with and without CAFs was performed using two‐way ANOVA with Fisher LSD post‐test, *P < 0.05, **P < 0.01. Lower panels: Light microscopy images of pancreatic, colon, and gastric cancer organoids, respectively, co‐cultured with CAFs 72 h post treatment with indicated drugs. Red arrows point toward the organoids while CAFs represent the lower, more dispersed layer of the culture. Scale bar, 50 μm. All photographs are representative to *n* = 3 experiment replicates.

For all three cancer types, the combination most effective in reducing organoid viability was carfilzomib (CFZ), VER‐155008, and nelfinavir (NLV). This combination proved to be significantly more potent than nab‐paclitaxel and gemcitabine in pancreatic cancer (*P* value < 0.0001), FOLFOX and FOLFIRI in colon cancer (*P* value < 0.05) and FOLFOX in GC (*P* value < 0.01) (Fig. 2A–C). Additionally, the calculated values of half maximal inhibitory concentration (IC50) for carfilzomib (CFZ), VER‐155008, and nelfinavir (NLV) were lower than those of tested chemotherapies (Fig. S2J–M). These tests allowed us to establish a platform to test and compare the sensitivity to both standard and experimental drug combinations for further development in this study.

To elucidate the effect of CAFs on effect of the tested anticancer therapies, we directly co‐cultured our established CAF lines with cancer organoids and subsequently conducted *in vitro* drug tests. In the co‐cultures, CAFs developed cell layers on top of which organoids reassembled, visibly separated from CAFs; in the case of GC organoids, CAFs grouped around the reassembled organoids (Fig. 2D–F, red arrows point toward organoids). The presence of CAFs had a protocol‐ and cancer‐specific effects on sensitivity to the treatment. Statistically significant effects of CAFs leading to increased resistance were observed in pancreatic cancer and colon cancer for specific standard‐of‐care protocols (Fig. 2D,E) and for two experimental protocols in GC (Fig. 2F). We calculated values of IC50 for nab‐paclitaxel + gemcitabine, FOLFOX and FLOT for monocultures and direct co‐cultures with CAFs in selected pairs of PDOs, which supported these results (Fig. S2K–M).

To check whether we would observe a similar paracrine effect of CAFs on cancer organoid viability, we co‐cultured cancer organoids with CAFs in a transwell system—CAFs were placed on the membrane of an insert, while organoids were placed at the bottom of the culture well (Fig. S3A). Using this approach, we performed drug tests for selected combinations of inhibitors and found that the paracrine effect of CAFs on those combinations did not significantly differ from that of the direct co‐culture (Fig. S3B–D).

These data suggest that the presence of CAFs influences anticancer treatments in PDOs, in general increasing the resistance of organoids to both clinically established and experimental protocols. Moreover, we confirmed that the paracrine effect of CAFs in our experimental setup appeared to be comparable to their direct effect.

### 3.3 Patient clinical outcomes in relation to *in vitro* organoid drug tests

We further used the biobank to investigate whether the clinical outcome of patients, whose tissues were used to derive organoids, matched our *in vitro* standard‐of‐care treatment data. The therapy outcome information was limited to a total of 19 out of 30 organoid donor patients due to therapy discontinuation caused by medical or patient‐led decisions (Fig. 1B–D). We evaluated both monocultures and co‐cultures with tissue‐matched CAFs to compare *in vitro* results with equivalent standard therapies in patients. Using this approach, we wanted to elucidate how well our biobank captured patient responses and whether this effect changed when CAFs were included in the assays.

The clinical data were divided into two groups: SD and PD following the Response Evaluation Criteria in Solid Tumors [24], based on the first diagnosis carried out in a patient upon completing a particular chemotherapy administration. The PD group included patients whose lesions increased by at least 20% in the sum of diameters, from the last CT scan while undergoing a specific standard‐of‐care treatment. The SD group included all the patients who did not qualify for the PD group: decrease or disappearance of lesions and no medically significant increase in the sum of lesion diameters between CT scans.

**Fig. 3:**
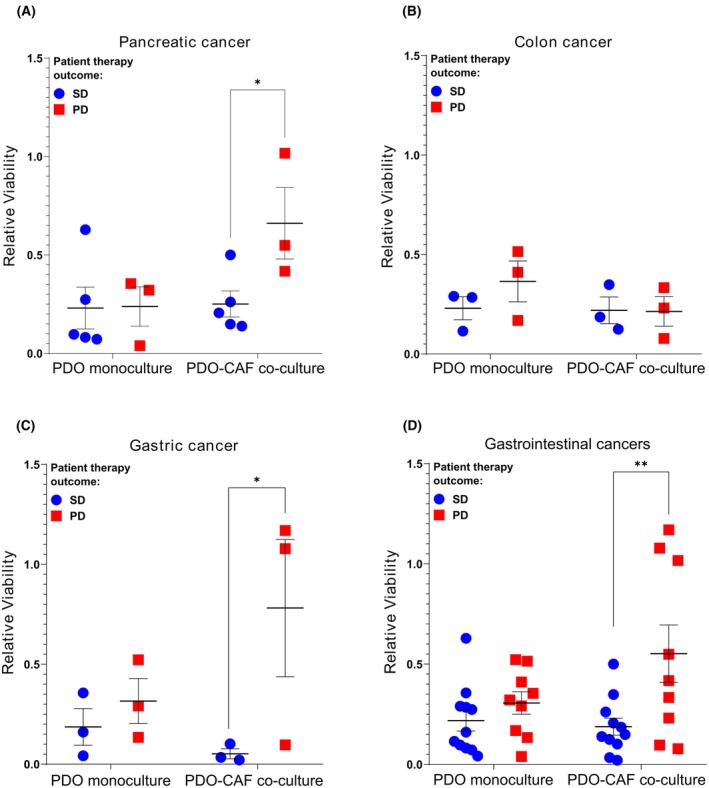
Comparison of patient clinical outcomes with results of *in vitro* organoid drug tests in the presence and absence of cancer‐associated fibroblasts (CAFs). (A–D) Pancreatic, colon, gastric, and gastrointestinal (all three types pooled together) cancer patient clinical outcomes for standard‐of‐care treatments compared with corresponding treatments conducted in *in vitro* cancer organoid monocultures or direct co‐cultures with CAFs. Relative viability results for the organoid drug tests were divided into groups: stable disease (SD in blue) and progressive disease (PD in red) representing clinical outcomes of each patient. Each symbol indicates the viability of a single tested organoid culture relative to the control; bars indicate means with standard error of the mean (SEM). Statistical analysis was performed using two‐way ANOVA with Fisher LSD post‐test, (*P < 0.05, **P < 0.01, ***P < 0.001). Each colored symbol indicates an independent biological replicate (for pancreatic cancer *n* = 8 individual treatments, for colon cancer *n* = 6 individual treatments, for gastric cancer *n* = 6 individual treatments) derived as an average of 3 technical replicates.

A statistically significant distinction between SD and PD outcomes was not observed in our organoid monocultures (Fig. 3A–D). For pancreatic and gastric cancers, but not for colon cancer, it became possible to discriminate between the groups when organoids were co‐cultured with CAFs (*P* value < 0.05) (Fig. 3A–C).

Given the limited number of patients with robust clinical data for each cancer type that lowered reliability of the analysis, we pooled the results for all three tested gastrointestinal cancer types to validate how well our entire biobank captures the patient response (Fig. 3D). Only in the case of co‐culture with CAFs, were we able to significantly discriminate between SD and PD outcomes (*P* value < 0.003) (Fig. 3D). A patient‐matched analysis within the separated SD and PD groups additionally revealed that the effect of CAF cells was significant only in the PD groups (Fig. S3E–H).

These data suggest that the inclusion of CAFs in analyses using viability readouts in PDO cultures has a decisive effect on improving their relevance to clinical outcomes in cancer patients. Extending the analysis to three cancer types, and consequently more samples, improves the statistical support for this result.

### 3.4 Organoid Convolution Assay determines tumor stage and predicts clinical outcomes of oncological patients

**Fig. 4:**
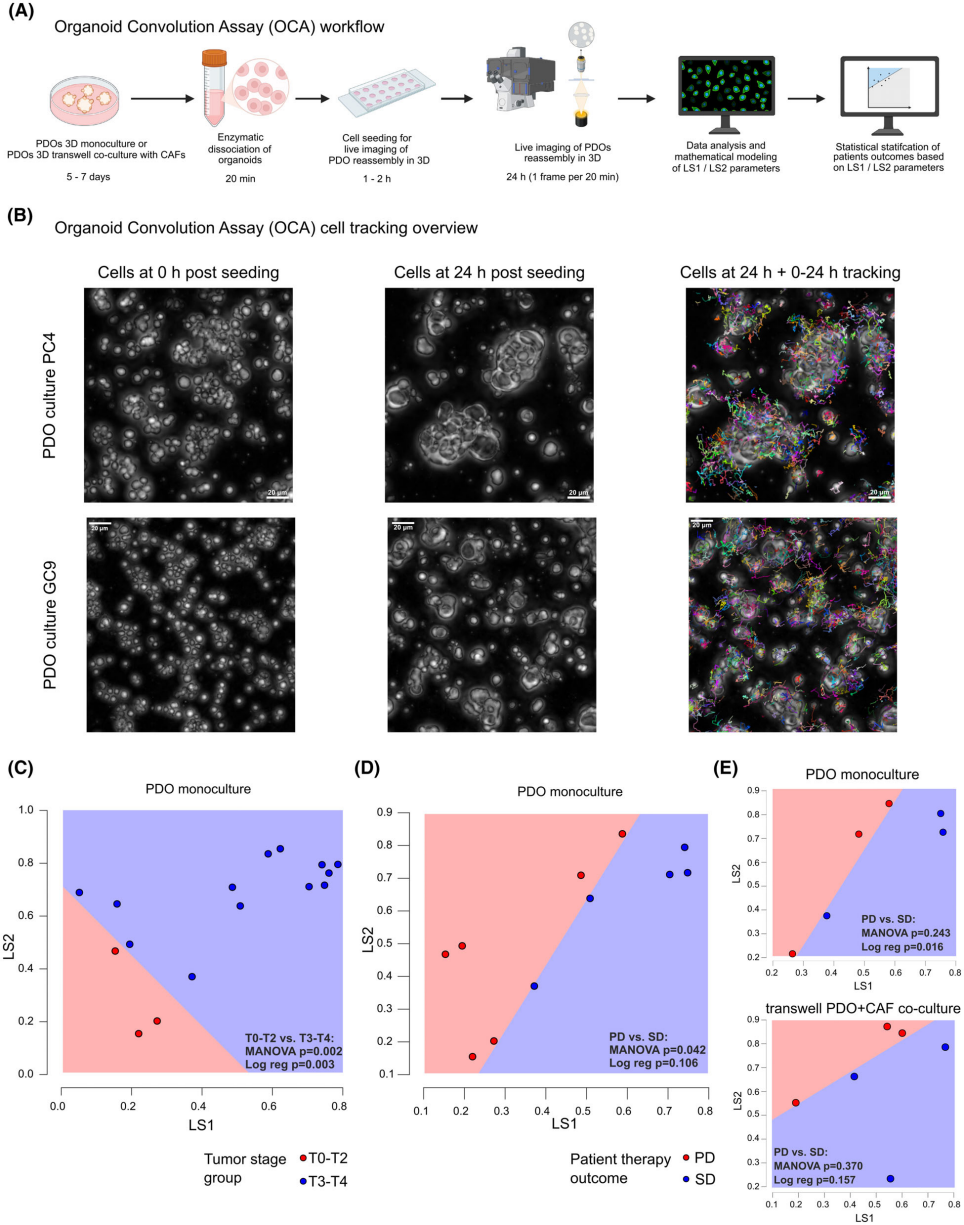
Organoid Convolution Assay (OCA) results allow for the determination of tumor stage and the prediction of clinical outcomes in cancer patients (A) Diagram showing the OCA flowchart, consisting of 3D patient‐derived cancer organoid culture (PDOs), specimen preparation, live‐cell imaging microscopy and data analysis, mathematical modeling and statistical stratification. (B) Examples of pancreatic cancer organoids (PC4) and gastric cancer organoids (GC9) bright‐field microscopy images at time point 0 h after seeding of cells derived from dissociated organoids (left panels), reassembled organoids at time point 24 h (middle panels) and cell/organoid tracking at the final 24 h time point; the cell movement paths are illustrated in different colors. The same field of view is presented in all panels. Scale bar, 20 μm. All photographs are representative to *n* = 3 experiment replicates. (C) Clustering of OCA results, which allowed for significant distinction of organoid‐derived data based on the tumor stage (T2 or lower tumor stage—marked in red, and T3–T4 tumor stage—marked in blue). Each dot represents the OCA result of a separate organoid culture from one of the three studied cancer types. The separation of groups was achieved using logistic regression classification to model the decision boundary matrix (shown as a boundary between two colors). The separation of the two populations was achieved with indicated *P* values via MANOVA (*P* = 0.002) and logistic regression tests (*P* = 0.003). Each circle indicates an independent patient sample (*n* = 16). (D) Clustering of OCA results, which allowed for significant distinction of organoid‐derived data based on the clinical outcomes of patients (stable disease—SD in blue and progressive disease—PD in red, when data were available for a given PDO). Each dot represents a separate patient sample (*n* = 11) from one of the 3 studied cancer types. The separation of groups was achieved using logistic regression classification to model the decision boundary matrix (shown as a boundary between two colors). The separation of the two populations was achieved with indicated *P* values via MANOVA (*P* = 0.042) and logistic regression tests (*P* = 0.106). (E) The OCA clustering was performed as in (D) for a limited set of organoid culture populations grown either as monocultures (upper panel) or in transwell co‐culture with cancer‐associated fibroblasts (CAFs) (lower panel). The separation of the two populations was achieved with indicated *P* values via MANOVA (*P* = 0.243 for monoculture and *P* = 0.370 for co‐culture) and logistic regression tests (*P* = 0.016 for monoculture and *P* = 0.157 for co‐culture). Each dot represents a separate patient‐derived organoid culture (*n* = 6)—with the same cultures +/−CAF shown in both panels. The separation of groups and statistical support were calculated as in (D). Data points in panels D and E (upper panel) represent subsets of the points shown in panel C, stratified according to different clinical criteria.

Considering the above results, we decided to test parameters other than viability to understand whether the tumor status and the therapy response in patients could be more efficiently predicted using organoid monocultures. This led to the development of a microscopy‐based methodology—the OCA (Fig. 4A).

Using this method, we observed dissociated organoid cells as they migrated, proliferated and grouped together to reestablish organoid structures. The first 24 h of the culture reassembly were recorded using bright‐field microscopy; then, the obtained time‐lapse images of the derived Z‐stacks were processed to track cells and assembling organoids (Fig. 4A,B, Movies S1– S3 and Table S5). This generated raw cell tracking data, which were then analyzed mathematically (Fig. S4A–C). We developed a set of parameters that described the process of reassembling organoids by dissociated cells (described in detail in Section 2.17 and Fig. S4A–C). These were used to calculate the LS1 and LS2 estimators, which can be visualized in a two‐dimensional coordinate system (Fig. 4C–E). The estimator values obtained for each tested sample (for which clinical data were available) were further stratified (by a decision boundary derived from logistic regression classification) and the support for the stratification was statistically tested (via MANOVA and logistic regression tests) for chosen sample parameters. A clear decision boundary was achieved for the stratification of tumor stages T0‐T2 vs. T3‐T4 groups, with *P* value of 0.002 using the MANOVA test (Fig. 4C). Interestingly, for a sample subset with available therapy outcome information, we were able to stratify the PD vs. SD patient outcome groups with *P* value of 0.042 via the MANOVA test, and again, a clear decision boundary was derived from logistic regression classification (Fig. 4D). Here, we limited the analysis to all cancer types pooled together, as sample numbers lower than 3 in each sample group were not suitable for the used statistical analysis.

Taking into account the influence of CAFs shown in Fig. 2, we decided to test whether co‐culturing organoids with CAFs would significantly alter the OCA results. To allow for unimpeded reassembly of organoid‐forming cells, this was performed in the CAF‐PDO indirect transwell co‐culture setup, shown earlier to perform similarly to direct co‐culture in drug tests (Fig. S2B–D). To this end, PDOs were cultured in the presence of CAFs (mediated by transwell) for a week, before conducting the OCA. For the six samples tested with and without CAFs, we obtained worse PD vs. SD stratification and statistical support in the presence of CAFs (*P* value 0.157 in logistic regression test and *P* value 0.370 for the MANOVA test) than in the absence of CAF (*P* value 0.016 in logistic regression test and *P* value 0.243 for the MANOVA test; Fig. 4E). This suggests that the accuracy of the OCA‐based stratification was not improved by the presence of CAFs, unlike the viability assays shown in Section 3.3.

Our newly developed OCA methodology monitors PDO assembly dynamics and mathematically derives information that is useful for patient diagnosis and outcome stratification. This approach shows that clinically relevant data beyond viability‐related parameters are preserved in the *in vitro* grown organoids. This information may be affected by the presence of the microenvironment (e.g., CAFs) differently than viability, and is potentially relevant for companion diagnostics using PDOs, as shown here for cancer patients.

## 4 Discussion

In this study, we sought to gradually refine clinically relevant drug testing methodologies in an organoid biobank derived from three digestive system cancer types: colon, pancreatic and gastric cancers. Ten selected, characterized organoid cultures from each of the three cancer types provided a sufficient population to compare the *in vitro* sensitivity to standard therapy regimens with selected experimental compound combinations in each cancer type. Throughout this study, the best‐performing combination in all the tested models proved to be carfilzomib, VER‐155008 and nelfinavir, first described previously by our group [18]. As carfilzomib and nelfinavir are both drugs already used clinically [31, 32], and since VER‐155008 appears to be well‐tolerated by mice [18, 33] and more robust HSP70 inhibitors are being clinically tested [34, 35], this combination appears to hold promise for further preclinical evaluation.

The initial stage of the study described above allowed us to establish an experimental system to compare the PDO sensitivity to both standard and experimental anticancer protocols in the presence or absence of patient‐derived CAFs as representatives of the cancer microenvironment. In our 3D organoid‐CAF co‐cultures, we observed a significantly increased resistance in two out of six standard‐of‐care chemotherapies across 3 cancer types. This heterogeneity was consistent with reports by other researchers. The presence of CAFs was previously shown to alter the effects of selected treatments, as demonstrated for gemcitabine and paclitaxel in pancreatic cancer cell lines [36]. Majety et al. and Marusyk et al. also reported a variable influence of CAFs on treatment efficacy in breast cancer‐CAF 3D co‐culture models [37, 38]. Furthermore, Schuth et al. and Liu et al. showed increased protection of organoids from the anticancer effects of several agents when in co‐culture with CAFs, although the effect was moderate and dependent on the parameters measured [14, 39]. We also confirmed that the transwell co‐culture system, which models the paracrine effect of CAF cells, had a statistically comparable effect to direct co‐culture, mirroring earlier results [36].

We further investigated two patient‐derived cancer organoid methodologies in the context of their ability to predict the clinical outcomes of patients. Our study had fewer samples with available therapy outcome information in each cancer type than several studies with significant stratification in the investigated cancers [9, 13], which reduced the significance of our results. However, the pooling of all our gastrointestinal tract cancer organoids, as shown in Fig. 3D, demonstrates that even in a sample population comparable to other studies only the presence of CAFs co‐cultured with cancer organoids leads to significant sample stratification, matching patient outcomes. It is important to note that, to our knowledge, there have been no publications directly comparing the performance of cancer organoids co‐cultured with CAFs versus organoid monocultures in modeling anticancer treatments responses in patients. The closest approach was published by Farin et al., who performed drug sensitivity assays and identified gene signatures associated with drug sensitivity in organoid cultures with and without CAFs. The authors compared the drug sensitivity results with sensitivity profiles of patients with matching colon cancer subtypes and correlated the gene expression signatures with database‐derived patient survival. In both cases, the co‐culture with CAFs improved the predictive potential of colon cancer organoids [13]. Our results simplify and supplement these findings by directly demonstrating an improvement in drug sensitivity matching between *in vitro* organoid‐CAF co‐cultures and donor patient outcomes.

Taking into account the above results and the fact that CAF cultures proved less successful to establish than the organoids, we sought a way to increase the accuracy of the correlation between organoid monocultures and the clinical outcomes of the donor patients. This led to the development of an alternative assay to standard PDO viability measurements—the OCA. In this assay, based on live‐cell imaging of the reassembly of organoid structures and mathematical modeling, we distinguished not only between low/moderate cancer stages (T0‐T2) vs. higher cancer stages (T3‐T4), but also between SD and PD clinical outcomes in donor patients, with no further significant alteration by the paracrine influence of CAFs. The assay results confirm that PDOs retain the histological and molecular properties of the original tumor responsible for tumor aggressiveness and drug sensitivity [9, 40, 41, 42]. Our results are also in line with those from other research groups which demonstrated that parameters such as morphology, invasion patterns, or expression signatures, can be successfully used to predict the clinical outcomes of patients in untreated *in vitro* models [43, 44, 45, 46].

The differential effect of CAF cells on PDO parameters other than viability is another dimension of our experimental system. Some studies by other researchers suggested that CAFs increase cancer cell migratory capabilities [47, 48, 49], while other studies suggested otherwise [50, 51, 52] or highlighted the complexity of the process [53]. Our results do not contradict these conclusions; while the OCA results for patient outcome stratification were not improved by the presence of CAFs. This is likely due to the fact that OCA results are not directly derived from cell migration alone, but instead integrate information on several organoid assembly parameters, which may be differently affected by CAFs.

The OCA approach is distinct from previous methodologies, which utilized microscopy‐based tools to harness the potential of organoids in advancing the development of new cancer models for more accurate drug discovery [54, 55, 56]. Our methodology is relatively simple, as it relies on multi‐plane, bright‐field microscopy, live‐cell imaging and open‐source software. This is also one of the limitations, as the current software does not perfectly segment cells and organoids and does not faultlessly detect multi‐cell structures. Therefore, the methodology could be further refined through dedicated software development.

Our study highlights the importance of increasing the complexity of organoid research models either by incorporating microenvironment‐derived cell populations (such as CAFs shown in this study) or by applying live‐structure assembly observation, mathematical modeling, and machine learning algorithms to acquire data that are usually omitted from the cancer‐derived *in vitro* cultures. Collectively, these approaches can result in more clinically relevant and personalized insights from patient‐derived material, which could ultimately help guide clinical decision‐making.

## 5 Conclusions

By testing across the biobank, we identified that an experimental triple‐combination therapy consisting of carfilzomib, VER‐155008, and nelfinavir was significantly more effective at reducing cell viability than current clinical standard‐of‐care regimens (such as FOLFIRINOX, FOLFOX, or FLOT) across all three digestive cancer types.

The inclusion of CAFs in co‐culture models demonstrated that the tumor microenvironment plays an important role in therapeutic response: CAFs selectively increased the resistance of organoids to both standard or experimental treatments, both direct contact and paracrine (indirect/transwell) signaling from CAFs provided similar levels of protection to the cancer cells.

When using standard (ATP‐based) viability assays, PDO monocultures failed to significantly distinguish between patients with SD and PD clinical outcomes. However, incorporating CAFs into the culture allowed us to significantly discriminate between these clinical outcomes, highlighting that the microenvironment is essential for PDOs to accurately mirror patient responses in viability tests.

This study introduced a novel, microscopy‐based methodology called the OCA, which tracks the dynamics of how dissociated cells reassemble into organoids. Unlike viability assays, the OCA approach allowed us to successfully stratify patients by tumor stage (T0‐T2 vs. T3‐T4) and clinical outcome (SD vs. PD).

By leveraging patient‐specific models, this study yields high‐fidelity insights that may serve as vital tools in tailoring individual treatment strategies.

## Conflict of interest

The authors declare no conflicts of interest.

## Author contributions

DW, MG, and LD contributed to the conceptualization and wrote the manuscript. DW, MG, LD, and MZ contributed to the methodology. DW, MG, LD, MJ, AG, TG, and WW contributed to the investigation. DW, MG and MJ contributed to the data curating and funding acquisition. WK, ML, TO, RS, and MM‐G contributed to the resources. DW, MG, LD, MJ, and AG contributed to the revisions. DW contributed to the supervision.

## Supporting information


**Fig. S1.** Histopathological characterization of donor tissues, cancer organoids, and CAF markers characterization.
**Data S1.** Supplementary Figures and Movie legends.
**Fig. S2.** Whole‐exome sequencing additional charts.
**Fig. S3.** Comparison of direct and indirect CAFs co‐culture with PDOs.
**Fig. S4.** Organoid Convolution Assay flow chart.
**Fig. S5.** Uncropped western blots.

**Table S1.** Pancreatic cancer organoids selected WES table.

**Table S2.** Colon cancer organoids selected WES table.

**Table S3.** Gastric cancer organoids selected WES table.

**Table S4.** Copy number variation selected WES table.

**Table S5.** LS1 and LS2 coordinates.

**Movie S1.** Organoid Convolution Assay movie of pancreatic cancer organoids.

**Movie S2.** Organoid Convolution Assay movie of gastric cancer organoids.

**Movie S3.** Organoid Convolution Assay movie of colon cancer organoids.

## Data Availability

Data underlying the results presented in this study are available in Supporting Information Tables and available upon request from the corresponding author, with restrictions in case of a sensitive human subject genomic data.
