# Supplementary material for: Patient therapy outcome modeling in cancer organoids is improved by cancer‐associated fibroblasts and organoid assembly convolution
Source: Mol Oncol. 2026 Jun 5;20(7):1694–712. doi: 10.1002/1878-0261.70282 (PMC13352955; doi:10.1002/1878-0261.70282)
Supplement: Supplementary file 1 — Fig. S1. Histopathological characterization of donor tissues, cancer organoids, and CAF markers characterization. Data S1. Supplementary Figures and Movie legends. Fig. S2. Whole‐exome sequencing additional charts. Fig. S3. Comparison of direct and indirect CAFs co‐culture with PDOs. Fig. S4. Organoid Convolution Assay flow chart. Fig. S5. Uncropped western blots. [file MOL2-20-1694-s009.zip › mol270282-sup-0014-Supplementary_Figure_Movie_legends.pdf]

**Supplementary Figure 1 Histopathological characterization of donor tissues, cancer organoids and CAF markers characterization. Related to Figure 1.**

**(A-C)** Hematoxylin-Eosin staining and diagnosis in pancreatic cancer (PC), colon cancer (CC) and gastric cancer (GC) respectively patients' samples used to generate organoid biobank. Pancreatic cancer samples for patients: PC1, PC2, PC5 and PC6 were already characterized by Oroń et al., 2022 [18] presented in Figure S3: PC1 is PDA9, PC2 is PDA12, PC5 is PDA10, PC6 is PDA11. Scale bar 200 µm. All photos are representative to standard histopathology patient diagnostic procedures.

**(D)** Cancer associated fibroblasts marker protein levels in pancreatic cancer CAFs line (PCAFs), gastric cancer CAFs line (GCAFs), colon cancer CAFs line (CCAFs) and normal skin fibroblasts (NormF) determined by western blot along cancer associated fibroblasts marker protein levels in pancreatic (PCO), colon (CCO) and gastric (GCO) cancer organoid lines determined also by western blot. M stands for molecular weight marker.

**(E-G)** Hematoxylin-Eosin staining in pancreatic cancer (PC), colon cancer (CC) and gastric cancer (GC) tumor organoids. Assessment of cancer organoid features: “malignant cells” refers to atypical cells with pleomorphism, hyperchromatic nuclei, high nuclear-to-cytoplasmic ratio, “disrupted organoid architecture” refers to irregular, disorganized 3D structures, “glandular type” (adenocarcinoma-like, including papillary pattern) refers to distorted glands or pseudo-papillary structures, “solid pattern” refers to compact sheets of cells, “diffuse-type pattern” refers to dispersed cells, often signet ring. Scale bar is 60 µm. All photos are representative to n=3 culture replicates.

**Supplementary Figure 2 Whole-exome sequencing additional charts. Related to Figure 1, 2 and 3.**

**(A)** Bar graph displaying distribution of variant classifications across all organoids’ samples for the chosen genes.

**(B)** Bar graph displaying summary of variant types across all organoids’ samples for the chosen genes.

**(C)** Bar graph displaying distribution of Single Nucleotide Variants (SNV) classified by base substitution type across all organoids’ samples for the chosen genes, with T>C and C>T transitions representing the dominant signatures.

**(D)** Column graph displaying distribution of variants per sample across all organoids’ samples for the chosen genes, summarizing their frequency.

**(E)** Boxplot showing the distribution of variants across all organoids’ samples for the chosen genes, summarizing their frequency.

**(F)** Bar graph displaying top 10 mutated genes across all organoids’ samples for the chosen genes, summarizing their frequency and distribution of genetic alterations among them. Analysis in graphs **(A-F)** is performed in all n=30 organoids described in Figure 1.

**(G-I)** Heatmaps of p-values showcasing significance of difference in relative viability between mutant and wild-type status of chosen genes in tested cohorts of **(G)** pancreatic, **(H)** colon and **(I)** gastric cancer PDO monocultures and PDO with CAF cocultures. Color indicates the direction of the effect, with higher saturation indicating lower p-values. Hierarchical clustering was performed to group data with similar profiles in the heatmaps. Analyses in graphs **(G-I)** are performed in 3 organoid groups of n=10 organoids for each indicated cancer type. The genes have been chosen based on the cancer driver census in

the TCGA and COSMIC databases as in Fig. 1 H-J. Single nucleotide variants (nonsense, missense, indels) and copy number variants (copy number increase, copy number decrease) were included. Statistical analysis was performed using Mann–Whitney U test, *p < 0.05.

**(J)** Titration sensitivity curves of selected organoids from indicated cancer types to the Cafilzomib + Nelflenavir + VER-155008 combination (total concentration at a steady molar ratio is shown on x-axis, derived from the concentration used in Fig. 2). Points are averages of measurements in 2 organoid cultures from each cancer type (n=2, each point is an average of 2 technical replicates) with indicated SD and fitted variable-slope model curves. IC50 results are indicated in the legend for curves representing each cancer type.

**(K-M)** Chemotherapy titration sensitivity curves of selected organoids from indicated cancer types, with or without direct co-culture with cancer-matched CAF cells. Points are averages of measurements in 2 organoid cultures from each cancer type (n=2, each point is an average of 2 technical replicates) with indicated SD and fitted variable-slope model curves. IC50 results are indicated for +CAF and no CAF-derived sensitivity curves in the legends, for each cancer type corresponding to the concentration range of the cancer-specific therapeutic protocol (total drug mix concentrations are shown on x-axes, with a steady molar ratio derived from Figure 2).

**Supplementary Figure 3 Comparison of direct and indirect CAFs co-culture with PDOs. Related to Figure 2 and 3.**

**(A)** Experimental flowchart of drug testing in direct PDOs co-culture with CAFs and indirect/transwell PDOs co-culture with CAFs.

**(B-D)** Comparison of relative viability results for pancreatic, colon and gastric cancer organoids respectively drug tests conducted using indirect/transwell system co-culture with CAFs and direct co-culture with CAFs. Each symbol indicates viability of a single patient-derived PDO  relative to the control (average of 3 technical replicates), with horizontal lines linking the same sample in an indirect/transwell and a direct CAF co-culture. Dashed lines indicate average, normalized solvent control levels. Statistical analysis of difference between sample-matched PDO populations with and without CAFs was performed using two-way ANOVA with Fisher LSD post-test, *p < 0.05, **p < 0.01.

**(E-H)** Pancreatic, colon, gastric and gastrointestinal (all three types pooled together) cancer patient clinical outcomes for standard-of-care treatments compared to corresponding treatments conducted in *in vitro* cancer organoid monocultures or direct co-cultures with CAFs. Relative viability results for the organoid drug tests were divided into groups: Stable Disease (SD in blue) and Progressive Disease (PD in red) representing clinical outcomes of each patient. Each symbol indicates viability of a single patient-derived PDO relative to the control (average of 3 technical replicates), with horizontal lines linking the same sample in a monoculture and a direct CAF co-culture. Statistical analysis of difference between sample-matched PDO populations with and without CAFs was performed using two-way ANOVA with Fisher LSD post-test, *p < 0.05, **p < 0.01.

**Supplementary Figure 4 Organoid Convolution Assay flow chart. Related to Figure 4.**

**(A)** Diagram showing the process of calculation of parameters: DAA, DV, DC.
**(B)** Diagram showing the process of calculation of ANND-S parameter.
**(C)** Diagram showing the process of calculation of LS1 and LS2 estimators.

**Supplementary Figure 5** Uncropped Western Blot related to Supplementary Figure 1D.

**Supplementary movie 1 Organoid Convolution Assay movie of pancreatic cancer organoids. Related to Figure 4**

A movie obtained via bright field microscopy (obtained via standard deviation processing of the Z-stack) showing migrating and proliferating pancreatic cancer (PC4) organoids and organoid cells during the first 24 h of organoid assembly. Scale bar 20 µm.

**Supplementary movie 2 Organoid Convolution Assay movie of gastric cancer organoids. Related to Figure 4**

A movie obtained via bright field microscopy (obtained via standard deviation processing of the Z-stack) showing migrating and proliferating gastric cancer (GC5) organoids and organoid cells during the first 24 h of organoid assembly. Scale bar 20 µm.

**Supplementary movie 3 Organoid Convolution Assay movie of colon cancer organoids. Related to Figure 4**

A movie obtained via bright field microscopy (obtained via standard deviation processing of the Z-stack) showing migrating and proliferating colon cancer (CC9) organoids and organoid cells during the first 24 h of organoid assembly. Movement tracks are shown in different colors. Cells and organoids are outlined in magenta. Scale bar 20 µm.
