# Supplementary material for: Patient therapy outcome modeling in cancer organoids is improved by cancer‐associated fibroblasts and organoid assembly convolution
Source: Mol Oncol. 2026 Jun 5;20(7):1694–712. doi: 10.1002/1878-0261.70282 (PMC13352955; doi:10.1002/1878-0261.70282)
Supplement: Supplementary file 1 — Fig. S1. Histopathological characterization of donor tissues, cancer organoids, and CAF markers characterization. Data S1. Supplementary Figures and Movie legends. Fig. S2. Whole‐exome sequencing additional charts. Fig. S3. Comparison of direct and indirect CAFs co‐culture with PDOs. Fig. S4. Organoid Convolution Assay flow chart. Fig. S5. Uncropped western blots. [file MOL2-20-1694-s009.zip › mol270282-sup-0001-FigureS1.pdf]

Supplementary Figure 1

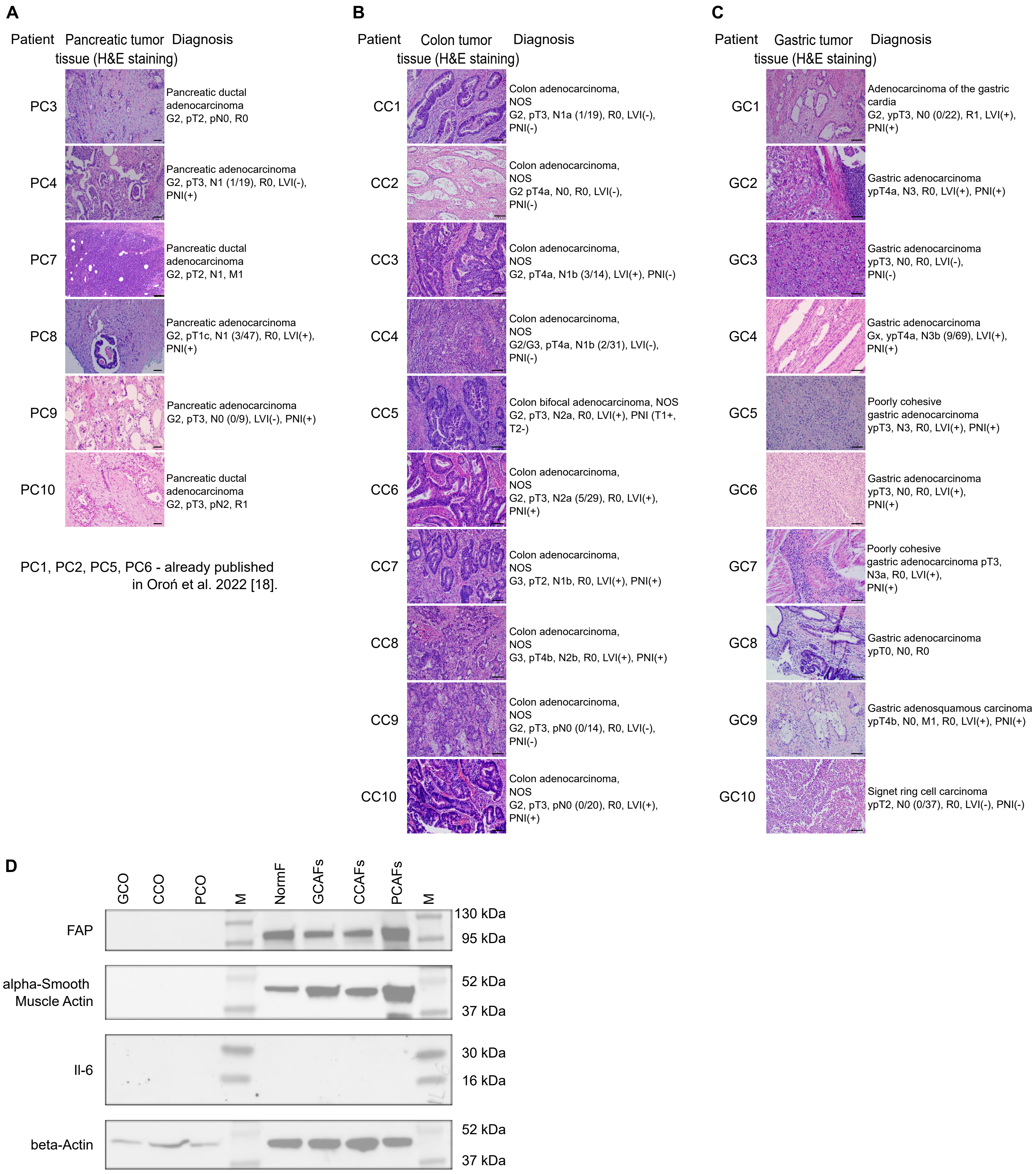

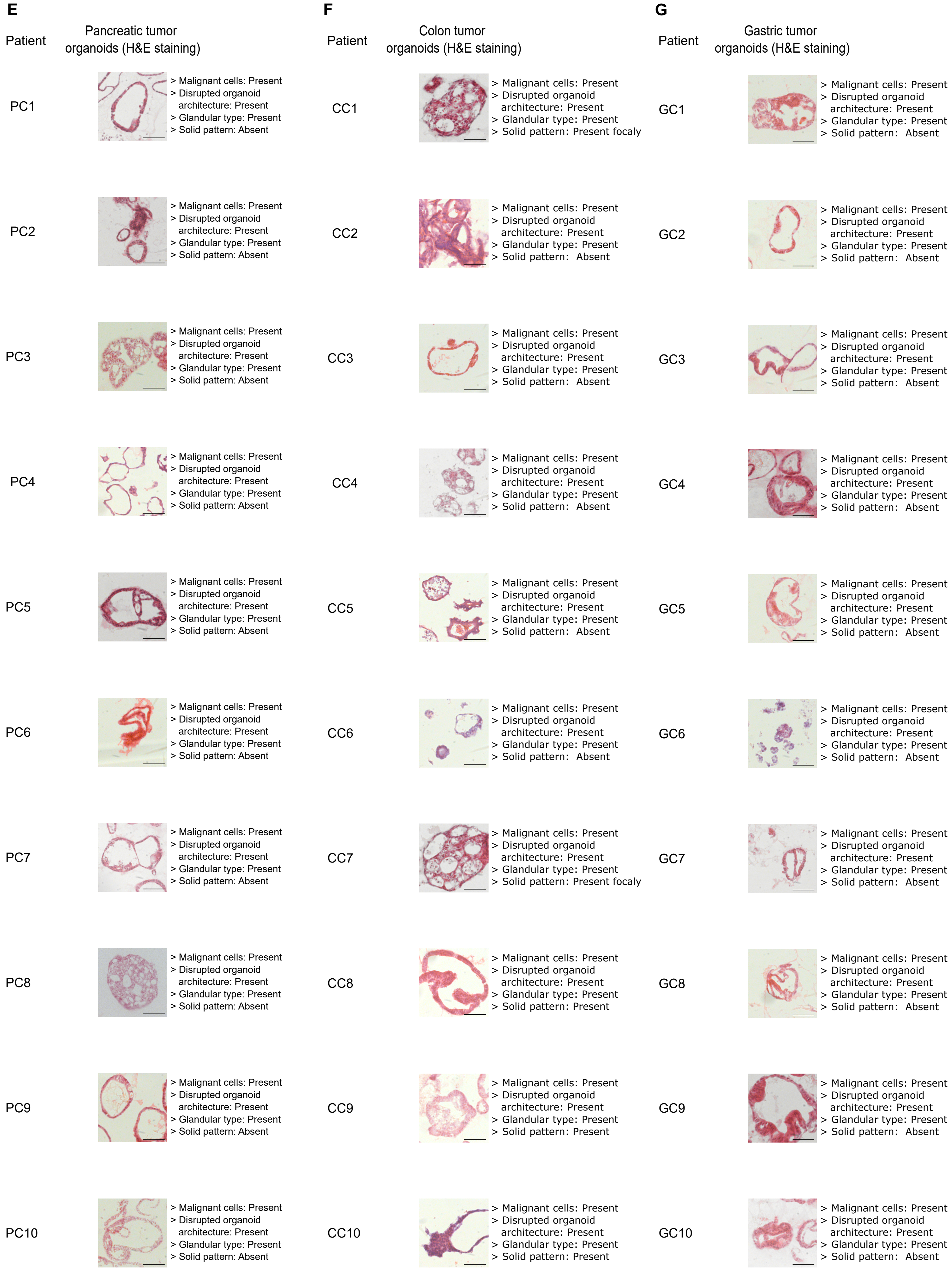
E
Patient
Pancreatic tumor organoids (H&E staining)
PC1
> Malignant cells: Present
> Disrupted organoid architecture: Present
> Glandular type: Present
> Solid pattern: Absent
PC2
> Malignant cells: Present
> Disrupted organoid architecture: Present
> Glandular type: Present
> Solid pattern: Absent
PC3
> Malignant cells: Present
> Disrupted organoid architecture: Present
> Glandular type: Present
> Solid pattern: Absent
PC4
> Malignant cells: Present
> Disrupted organoid architecture: Present
> Glandular type: Present
> Solid pattern: Absent
PC5
> Malignant cells: Present
> Disrupted organoid architecture: Present
> Glandular type: Present
> Solid pattern: Absent
PC6
> Malignant cells: Present
> Disrupted organoid architecture: Present
> Glandular type: Present
> Solid pattern: Absent
PC7
> Malignant cells: Present
> Disrupted organoid architecture: Present
> Glandular type: Present
> Solid pattern: Absent
PC8
> Malignant cells: Present
> Disrupted organoid architecture: Present
> Glandular type: Present
> Solid pattern: Absent
PC9
> Malignant cells: Present
> Disrupted organoid architecture: Present
> Glandular type: Present
> Solid pattern: Absent
PC10
> Malignant cells: Present
> Disrupted organoid architecture: Present
> Glandular type: Present
> Solid pattern: Absent
F
Patient
Colon tumor organoids (H&E staining)
CC1
> Malignant cells: Present
> Disrupted organoid architecture: Present
> Glandular type: Present
> Solid pattern: Present focaly
CC2
> Malignant cells: Present
> Disrupted organoid architecture: Present
> Glandular type: Present
> Solid pattern: Absent
CC3
> Malignant cells: Present
> Disrupted organoid architecture: Present
> Glandular type: Present
> Solid pattern: Absent
CC4
> Malignant cells: Present
> Disrupted organoid architecture: Present
> Glandular type: Present
> Solid pattern: Absent
CC5
> Malignant cells: Present
> Disrupted organoid architecture: Present
> Glandular type: Present
> Solid pattern: Absent
CC6
> Malignant cells: Present
> Disrupted organoid architecture: Present
> Glandular type: Present
> Solid pattern: Absent
CC7
> Malignant cells: Present
> Disrupted organoid architecture: Present
> Glandular type: Present
> Solid pattern: Present focaly
CC8
> Malignant cells: Present
> Disrupted organoid architecture: Present
> Glandular type: Present
> Solid pattern: Present
CC9
> Malignant cells: Present
> Disrupted organoid architecture: Present
> Glandular type: Present
> Solid pattern: Present
CC10
> Malignant cells: Present
> Disrupted organoid architecture: Present
> Glandular type: Present
> Solid pattern: Present
G
Patient
Gastric tumor organoids (H&E staining)
GC1
> Malignant cells: Present
> Disrupted organoid architecture: Present
> Glandular type: Present
> Solid pattern: Absent
GC2
> Malignant cells: Present
> Disrupted organoid architecture: Present
> Glandular type: Present
> Solid pattern: Absent
GC3
> Malignant cells: Present
> Disrupted organoid architecture: Present
> Glandular type: Present
> Solid pattern: Absent
GC4
> Malignant cells: Present
> Disrupted organoid architecture: Present
> Glandular type: Present
> Solid pattern: Absent
GC5
> Malignant cells: Present
> Disrupted organoid architecture: Present
> Glandular type: Present
> Solid pattern: Absent
GC6
> Malignant cells: Present
> Disrupted organoid architecture: Present
> Glandular type: Present
> Solid pattern: Absent
GC7
> Malignant cells: Present
> Disrupted organoid architecture: Present
> Glandular type: Present
> Solid pattern: Absent
GC8
> Malignant cells: Present
> Disrupted organoid architecture: Present
> Glandular type: Present
> Solid pattern: Absent
GC9
> Malignant cells: Present
> Disrupted organoid architecture: Present
> Glandular type: Present
> Solid pattern: Absent
GC10
> Malignant cells: Present
> Disrupted organoid architecture: Present
> Glandular type: Present
> Solid pattern: Absent
