# Supplementary material for: Patient therapy outcome modeling in cancer organoids is improved by cancer‐associated fibroblasts and organoid assembly convolution
Source: Mol Oncol. 2026 Jun 5;20(7):1694–712. doi: 10.1002/1878-0261.70282 (PMC13352955; doi:10.1002/1878-0261.70282)
Supplement: Supplementary file 1 — Fig. S1. Histopathological characterization of donor tissues, cancer organoids, and CAF markers characterization. Data S1. Supplementary Figures and Movie legends. Fig. S2. Whole‐exome sequencing additional charts. Fig. S3. Comparison of direct and indirect CAFs co‐culture with PDOs. Fig. S4. Organoid Convolution Assay flow chart. Fig. S5. Uncropped western blots. [file MOL2-20-1694-s009.zip › mol270282-sup-0002-FigureS2.pdf]

Supplementary Figure 2

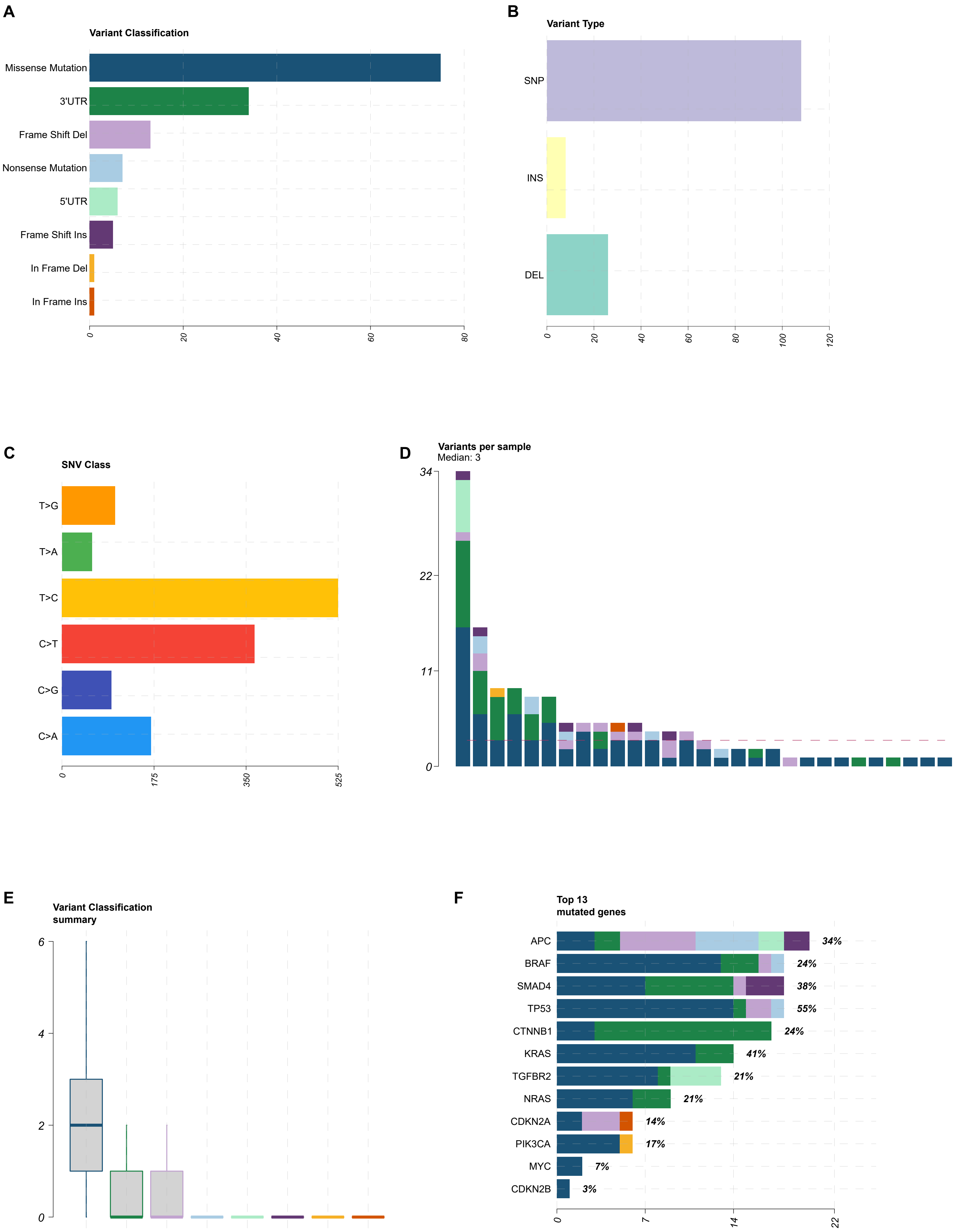

G

Pancreatic cancer

Clustered Drug-Gene Mutation Association Matrix

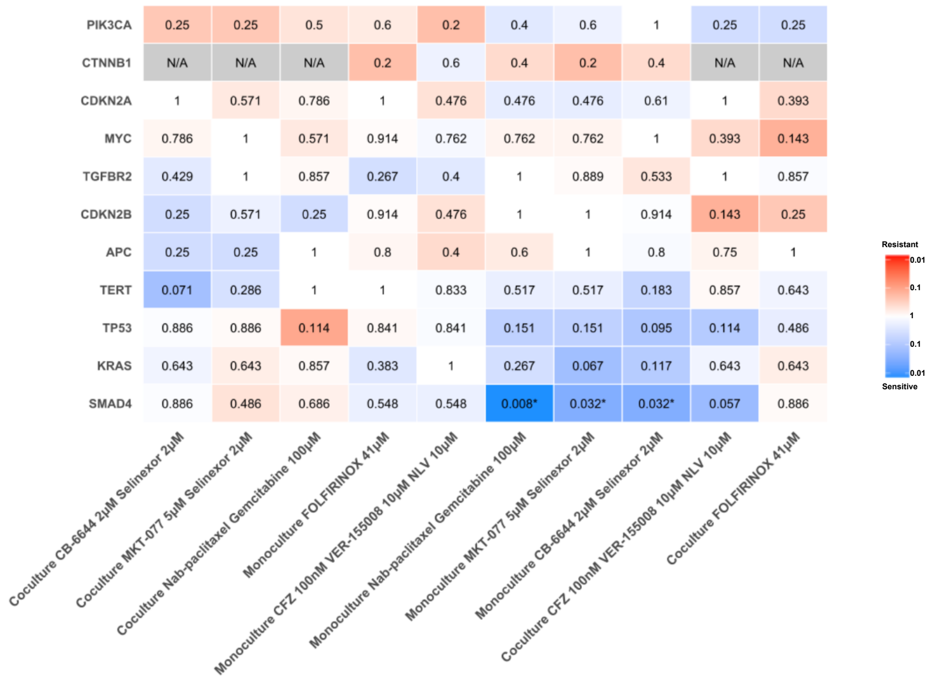

H

Colon cancer

Clustered Drug-Gene Mutation Association Matrix

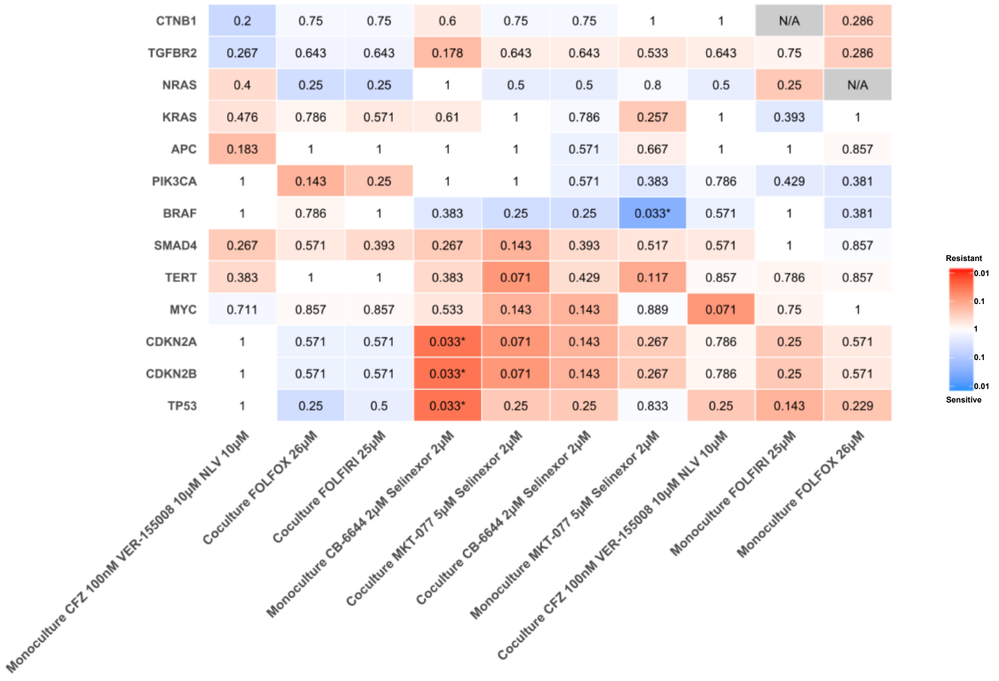

I

Gastric cancer

Clustered Drug-Gene Mutation Association Matrix

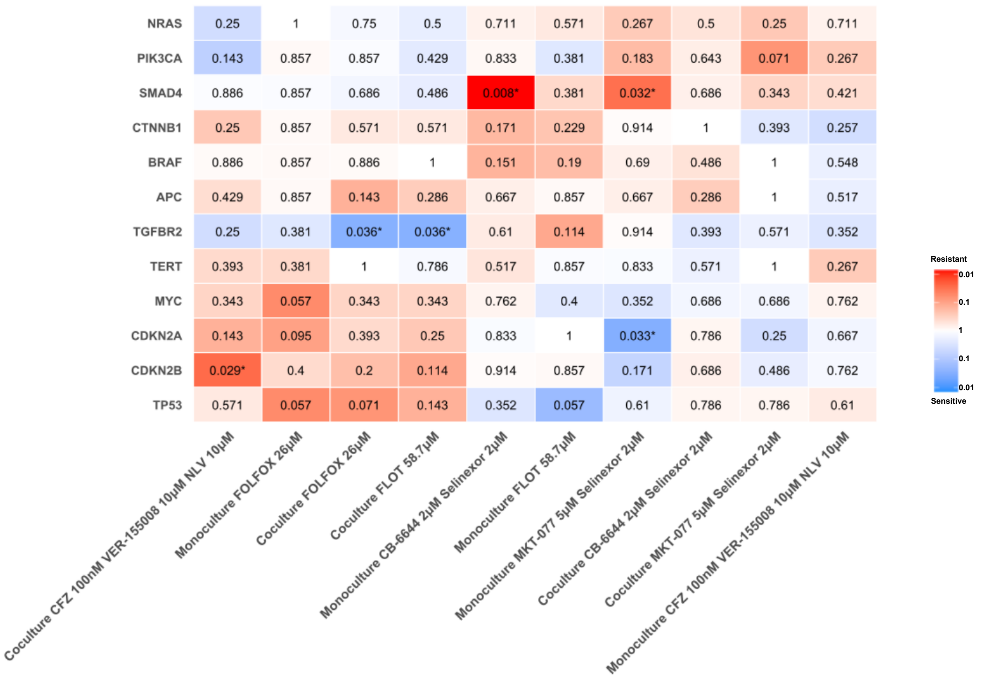

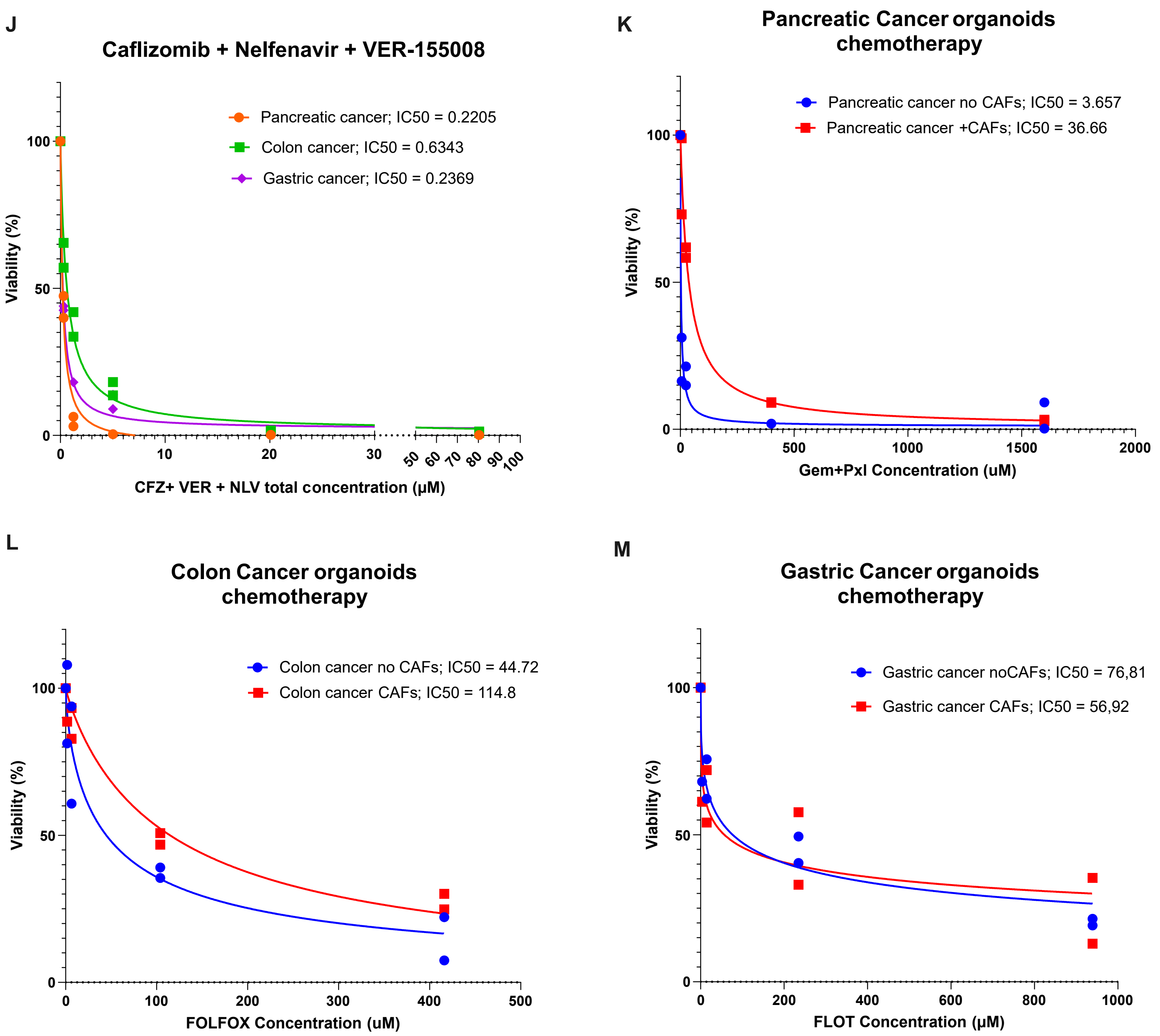
J
Caflizomib + Nelfenavir + VER-155008
Pancreatic cancer; IC50 = 0.2205
Colon cancer; IC50 = 0.6343
Gastric cancer; IC50 = 0.2369
Viability (%)
100
50
0
0
10
20
30
50
60
70
80
90
100
CFZ+ VER + NLV total concentration (μM)
K
Pancreatic Cancer organoids chemotherapy
Pancreatic cancer no CAFs; IC50 = 3.657
Pancreatic cancer +CAFs; IC50 = 36.66
Viability (%)
100
50
0
0
500
1000
1500
2000
Gem+Pxl Concentration (uM)
L
Colon Cancer organoids chemotherapy
Colon cancer no CAFs; IC50 = 44.72
Colon cancer CAFs; IC50 = 114.8
Viability (%)
100
50
0
0
100
200
300
400
500
FOLFOX Concentration (uM)
M
Gastric Cancer organoids chemotherapy
Gastric cancer noCAFs; IC50 = 76,81
Gastric cancer CAFs; IC50 = 56,92
Viability (%)
100
50
0
0
200
400
600
800
1000
FLOT Concentration (μM)
