# Supplementary figures and images for: Patient therapy outcome modeling in cancer organoids is improved by cancer‐associated fibroblasts and organoid assembly convolution
Source: Mol Oncol. 2026 Jun 5;20(7):1694–712. doi: 10.1002/1878-0261.70282 (PMC13352955; doi:10.1002/1878-0261.70282)

Supplementary figure 4

A

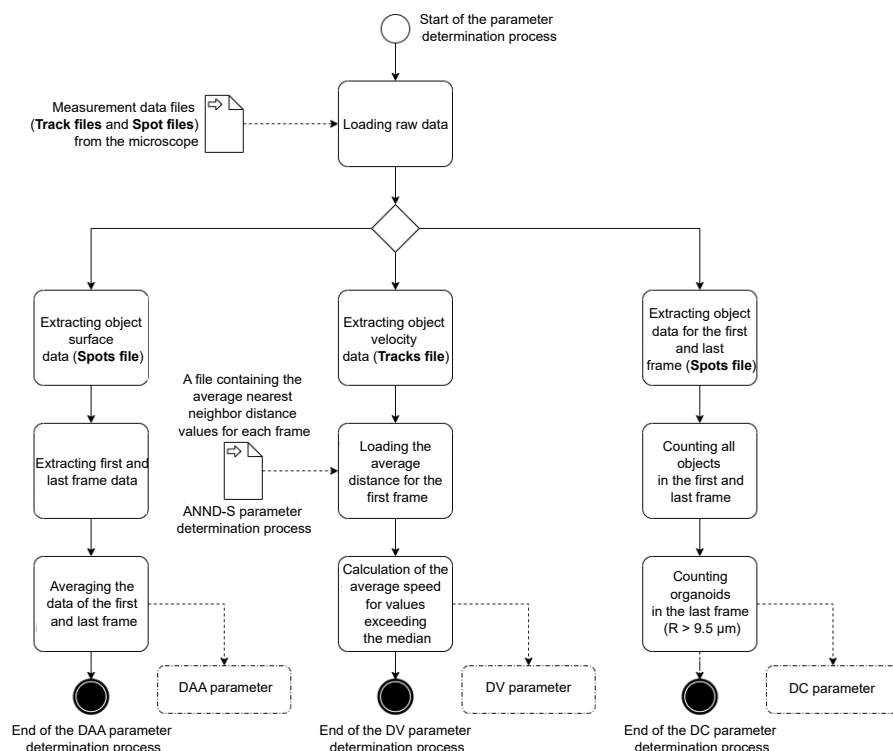

B

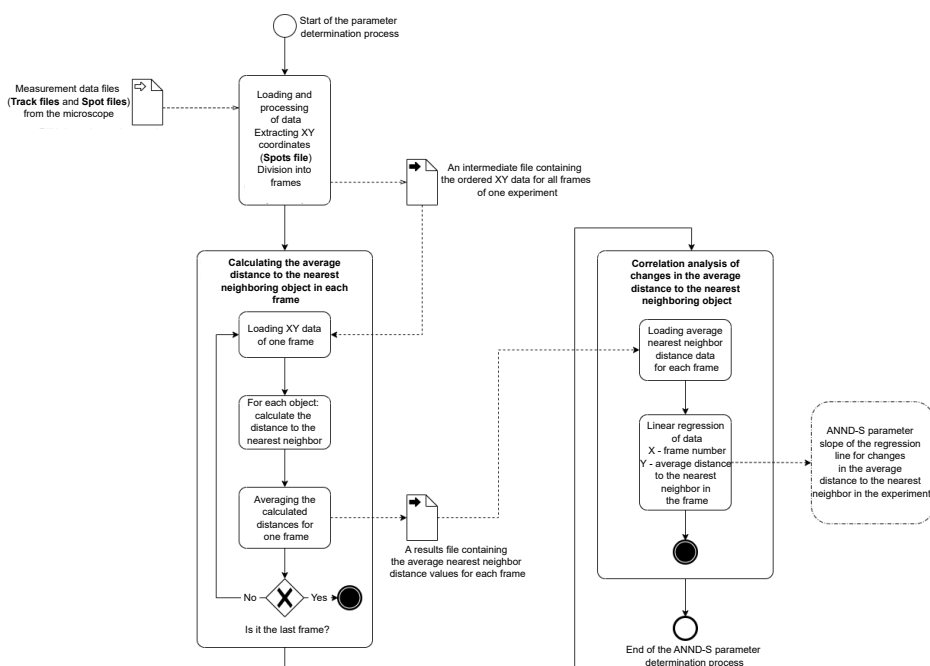

C

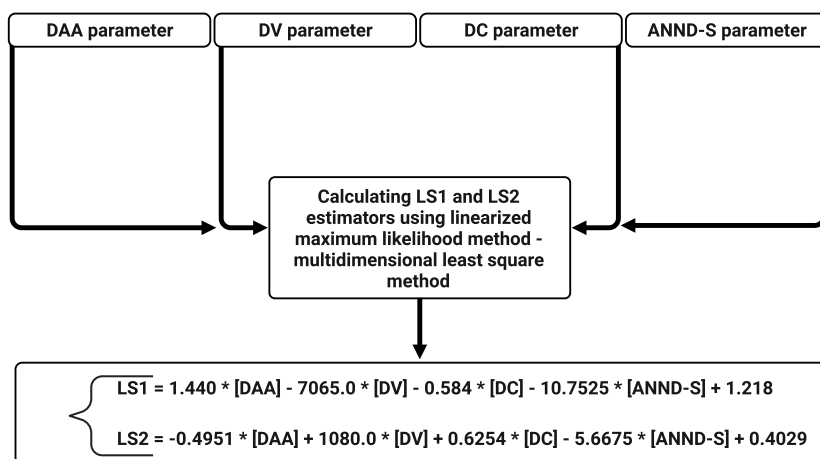

Supplement: Supplementary file 1 — Fig. S1. Histopathological characterization of donor tissues, cancer organoids, and CAF markers characterization. Data S1. Supplementary Figures and Movie legends. Fig. S2. Whole‐exome sequencing additional charts. Fig. S3. Comparison of direct and indirect CAFs co‐culture with PDOs. Fig. S4. Organoid Convolution Assay flow chart. Fig. S5. Uncropped western blots. [file MOL2-20-1694-s009.zip › mol270282-sup-0004-FigureS4.pdf]

**A**

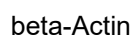

Supplement: Supplementary file 1 — Fig. S1. Histopathological characterization of donor tissues, cancer organoids, and CAF markers characterization. Data S1. Supplementary Figures and Movie legends. Fig. S2. Whole‐exome sequencing additional charts. Fig. S3. Comparison of direct and indirect CAFs co‐culture with PDOs. Fig. S4. Organoid Convolution Assay flow chart. Fig. S5. Uncropped western blots. [file MOL2-20-1694-s009.zip › mol270282-sup-0005-FigureS5.pdf]
